# COVID-19. An update for orthopedic surgeons

**DOI:** 10.1051/sicotj/2020022

**Published:** 2020-07-01

**Authors:** Mohammad Kamal Abdelnasser, Mohamed Morsy, Ahmed E. Osman, Ayman F. AbdelKawi, Mahmoud Fouad Ibrahim, Amr Eisa, Amr A. Fadle, Amr Hatem, Mohammed Anter Abdelhameed, Ahmed Abdelazim A. Hassan, Ahmed Shawky Abdelgawaad

**Affiliations:** 1 Orthopedic Department, Assiut University Hospitals Assiut 71515 Egypt; 2 Spine center, Helios Klinikum Erfurt Nordhaeuser street 74 88089 Erfurt Germany

**Keywords:** COVID-19, Orthopaedics

## Abstract

The COVID-19 pandemic has affected our world in a short period of time, and the orthopedic surgery practice was not an exclusion. Elective care was deferred in most health care facilities and emergency care was continued with strict precautions. With rapid progression of the pandemic, the response of the medical community is also rapidly changing in all aspects of delivering care. This led to a large number of publications with reports, guidelines, measures, ways to react to the crisis, and post-pandemic predictions and speculations. In this review we aimed at summarizing all the relevant information to the orthopedic surgery community. To do this, a comprehensive search was performed with all related terms on two scientific search engines, PubMed and SCOPUS, and the results were filtered by the Preferred Reporting Items for Systematic Reviews and Meta-Analyses (PRISMA) method. The result was 72 articles that were further reduced to 33 articles after full text reading. The resultant information was organized under 5 main headings; the impact of pandemic on the orthopedic practice, COVID-19 and the trauma patient, elective and emergency surgeries during the pandemic, peri-operative management of the patient with COVID-19, Miscellaneous effects of the pandemic such as those on training programs and the evolution of telemedicine. This review represents the most up to date information published in the literature that is a must-know to every orthopedic surgeon.

## Introduction

COVID-19 or SARS-CoV-2 was first identified as a potential infectious threat in China in December 2019, [1–3] and declared as a pandemic by the World Health Organization on March 11, 2020 [4]. With the massive burden on health systems around the world, COVID-19 has heavily impacted all aspects of the medical practice including specialities that are not directly related to its clinical effects such as orthopedic surgery. Elective surgical procedures have been postponed in order to reduce the burden on health systems and allow for more availability of hospital beds for the more needy. Management of emergent and urgent surgical cases has also been affected [5]. A continuous need is present to address the daily new information and to employ them in our orthopedic practices. Moreover, with more countries reaching their peak and plateau phase, healthcare facilities are getting ready to reopen and resume medical care. This will require a solid understanding of the precautions required for this resumption during such a critical phase, which may extend for a few months ahead, not to mention some speculations of a second wave of COVID-19 infection in the near future. We aimed at delivering a comprehensive review summarizing the most recent information and guidelines relevant to the orthopedic community available in the literature to help us plan for the current phase and those yet to come.

## Methods

**Figure 1:**
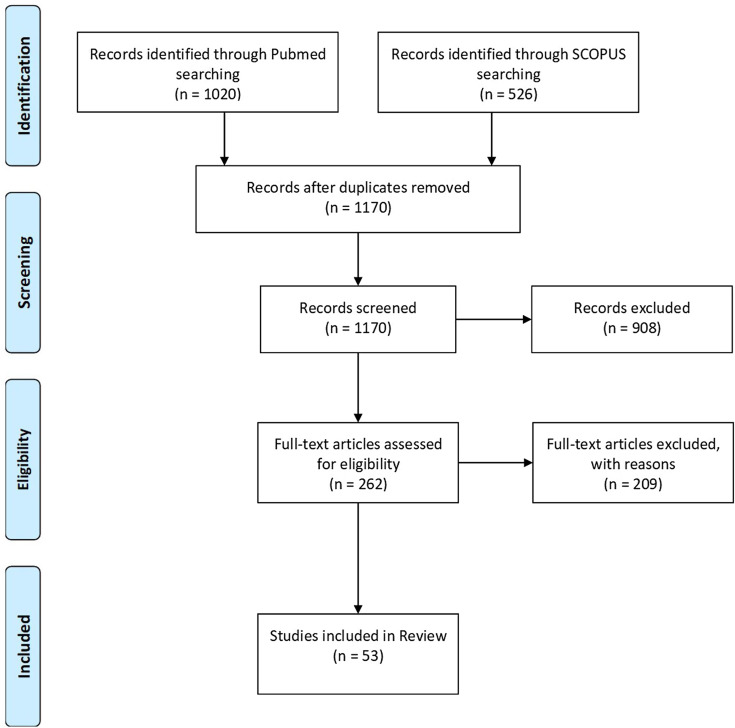
The PRISMA flow chart employed showing the article selection process for the review.

To provide the most relevant and up to date information for the orthopedic community, a systematic approach was used to gather information. A literature search was conducted on May 22nd on Medline and SCOPUS with the terms “COVID-19”, “COVID 19”, “COVID”, “Corona virus” or “Corona”, together with “orthopedic”, “orthopaedic”, “orthopedics”, “orthopaedics”, “surgery”, and “surgical” including all possible combinations. The Preferred Reporting Items for Systematic Reviews and Meta-Analyses (PRISMA) method and flowchart were used to filter the results of the search (Figure 1) [6]. The search retrieved a total of 1546 articles, which were reduced to 1170 after omitting duplicates. Screening by title and abstract further reduced the number to 262 after exclusion of non-English language articles and those addressing details not relevant to the orthopedic specialty. The full texts of these articles were read, and 209 articles were further excluded that lacked relevant information. Relevant information was digested and organized under 5 main headings; the impact of COVID-19 pandemic on the orthopedic practice, COVID-19 and the trauma patient, elective and emergency surgeries during the pandemic, peri-operative management of the patient with COVID-19, Miscellaneous effects of the pandemic such as those on training programs and the evolution of telemedicine.

### The impact of COVID-19 pandemic

Lack of sufficient evidence and the highly contagious nature of COVID-19 led to drastic measures implemented by many countries varying from social distancing to total lockdown, which had tremendous global economic and social effects [7, 8]. The COVID-19 pandemic represents an unprecedented challenge to healthcare systems mainly due to the exponential expansion of the patient population in need of hospitalization surpassing available resources [9–14]. With the risks posed by shortage of Personal Protective Equipment (PPE) [13–15] non-traditional solutions were developed such as 3D printed face shields, reusable gowns, and protocols for the re-use of PPE [18]. Moreover, the sudden overload of healthcare systems mandated institution of new hospitals as well as changing the bed capacity to increase respiratory care beds; this was coupled with initial reduction and later cancelation of all elective procedures in order to save the available resources and limit spread of the virus [19–27]. In the midst of this crisis, the orthopedic surgeon was surely affected: from de-specialization and serving on the frontline, to upgrades from fellow to faculty and reassignment of residents to clinical care rotations [18–28]. Managing COVID-19 patients with surgical emergencies and trauma with the risk of self-infection have led to a higher degree of anxiety and depression [32]. With the mandatory decrease in face-to-face encounters, online communication systems have flourished to provide meetings for faculty, quick and wide spread of knowledge, and outreach to patients through telehealth systems [18–21].

### COVID-19 and the trauma patient

So far, very few studies reported the association between COVID-19 and trauma patients [33–35]. Given the fact that patients with fractures especially of the lower extremity and those with limited ambulatory capacity are more susceptible to respiratory infection [36], the association between COVID-19 and trauma patients is not unlikely.

Mi et al. reported on 10 trauma patients with COVID-19, seven of them (70%) had a nosocomial infection after admission to the hospital because of their fracture [34]. Although SARS-CoV-2 was positive in only 6 patients, the characteristic CT ground-glass opacities were evident in all patients. Clinical symptoms were not different from those present in patients without factures. Lymphopenia was more common in patients with fractures. Moreover, D-Dimer and the median neutrophilic count were higher than the upper normal limits of the corresponding indicators. These might be special laboratory indicators of fractures in patients with COVID-19. Four patients (40%) died and three others (30%) developed severe pneumonia. The authors concluded that the association between COVID-19 pneumonia and fractures can lead to severe adverse outcomes and increased mortality [34].

Catellani et al. reported on 16 patients of proximal femoral fractures positive for COVID-19 [35]. All patients presented with fever and oxygen desaturation on ambient air; 14 of them required respiratory support. Improved respiratory parameters were evident in 12 out of 13 patients who underwent early fracture stabilization. The authors concluded that early fixation may contribute to the overall patient stability, improvement in physiological ventilation, seated mobilization, and general patient comfort in bed [35].

Nevertheless, the association between COVID-19 characteristic CT picture and trauma patients has also been reported in absence of symptoms related to COVID-19 pneumonia [33].

### Emergent, urgent, and elective procedures

The necessity to choose which operations to proceed with and which can wait is a challenging and sometimes difficult decision during the pandemic crisis. In the light of the available literature, this review will try to address the most relevant questions to our practice.

#### A. What is the definition of an elective procedure?

In the time of the pandemic, it is important to identify elective procedures or in other terms, the ones that could be delayed. Although this may sound simple, the pandemic itself has made such a sharp distinction impossible, creating a large gray zone. This question is particularly relevant to the orthopedic practice as 47% of the expenditure is from elective surgeries [37]. With no consensus reached in the orthopedic community, some authors recommended that this should be individualized to each facility according to its resources and to each patient according to the condition [37–39].

The Ohio Hospital Association (OHA) defined elective surgeries as those not meeting the following criteria “threat to the patient’s life if surgery or procedure is not performed, threat of permanent dysfunction of an extremity or an organ system, risk of metastasis or progression of staging, or risk of rapidly worsening to severe symptoms” [37, 38]. Patients with stable diseases (low or moderate risk of clinical deterioration) can be postponed, while patients with unstable disease (risk of short-term clinical deterioration) should be considered for surgery with precautions [40].

The COVID-19 status of the patient whether positive, negative, or not tested is another important factor that affects the time of surgical intervention [40].

#### B. Why postpone elective procedures?

Reducing surgeries saves resources including hospital beds, PPEs, as well as protecting the surgical staff [5]. This can also diminish the risk of perioperative complications and mortality, [40, 41] reduce unnecessary patient traffic and decrease the introduction and spread of disease among patients and health care providers [5].

#### C. What should be offered to patients as an alternative to surgical intervention?

Delays of operative intervention in elective cases although temporary, might extend for months as a best estimate. Patients should be offered sound alternatives to assist them bear the anticipated waiting times, this could be in the form of optimized medical treatment, individualized non-surgical options through multidisciplinary approaches, supportive online counseling, psychological support, and in pediatric patients engaging families and stressing on safety measures [40–42].

#### D. Are there any recommendations on categorizing and managing orthopedic conditions?

Many articles have tried to categorize various conditions according to urgency, [37, 38] as well as guidelines put forth by international societies [45–47].

Awad et al. stratified orthopedic conditions into five categories according to urgency, A through E, A being the most urgent [45]. Open fractures, acute neurovascular derangements as well as acute infections were rendered as emergent (A) to be operated within 24 h. Closed fractures were grouped under B or C. Deformities, arthroplasty and trigger finger were grouped under E [45]. Table 1 gives relevant examples to these recommended categories.

**Table 1:** Examples of priority stratification according to Massey et al. [39].

Subspecialty	Priority A	Priority B	Priority C	Priority D	Priority E
e.g., Spine	Closed reduction of a cervical Facet dislocation	Cauda equina syndrome		Operative lumbar discectomy with radiculopathy	Spondylolisthesis
Foot and ankle surgery			Operative foot fractures		Ankle arthroplasty or fusion

Farrell et al. suggested some management plans for the pediatric orthopedic patient [42]. In the pediatric trauma patient, modifications to standard care were mostly to the follow-up instructions and methods. Table 2 demonstrates two examples. As for elective orthopedic patient, the authors advocated either postponing the surgery or doing a minimally invasive procedure if feasible. Table 3 gives examples to these situations.

**Table 2:** Examples for Recommendations for Orthopedic Pediatric Trauma Management during COVID-19 pandemic [37].

Injury	What to do immediately	Follow-up
Gartland 1 Supracondylar fracture	Collar and cuff, removed by family at 3 weeks.	None required.
Fibular Fracture	Apply walking boot	Family to remove boot at week 4
	Weight bear as tolerated	Teleconference week 6

**Table 3:** Examples for Recommendations for Orthopedic Pediatric Elective Management during COVID-19 pandemic [37].

Example	Management	Rationale	Follow-up
New case of a club foot	Do not start a Ponseti casting	Ponseti method requires frequent reviews, risk of transmission	Ponsetti casting can be commenced later
			Review after COVID pandemic (3 months)
			Consider teleconferencing
Anterior Cruciate Ligament	Postpone	Excellent results can be still obtained with a period of delay	Follow-up after the pandemic.
			Offer prehab program.

Donnally et al. published on triaging patients with spine pathologies according to the Rothman Institute Guidelines during the COVID-19 era [48]. Patients were classified into three levels according to the urgency of surgical intervention and the facility in which the patient should be operated upon. Table 4 describes relevant examples for each level.

**Table 4:** Examples of triaging spine surgery in the COVID-19 era [44].

Level	Surgical Spine Pathologies	Recommendations
Level 1	Cervical or thoracic myelopathy (symptomatic; disc herniations, infections, tumor burden)	Proceed with surgical intervention at hospital location.
Level 2	Acute or subacute lumbar disc herniations (up to 6 weeks) with intractable pain	Proceed with surgical intervention at ambulatory surgical center (ASC) versus consider at hospital facility if low COVID-19 census.
Level 3	Compression fracture (without neurologic deficits)	Defer surgery or reconsider risks versus benefits of continued conservative management.
		Consider course of steroid therapy (injection or oral).
		Odontoid fractures in elderly will be managed conservatively, with option of treating symptomatic nonunion surgically in future.

#### E. How to triage patients during the COVID-19 pandemic?

**Figure 2:**
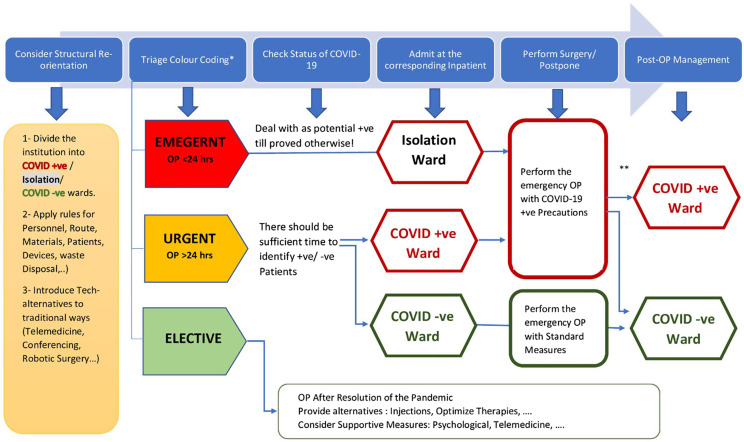
Suggested Flowchart for dealing with orthopaedic and Trauma cases during the period of the COVID-19 Pandemic. *Defining the urgency of the condition is multifactorial and differs according to the institution, resources and patient characteristics (see text). **The emergency patients stay postoperatively in the Isolation Ward till available test results for COVID-19 Infection.

Awad et al. recommended regional organization by assigning designated hospitals with orthopedic staff to treat only suspected or confirmed COVID-19 patients and other hospitals in the same regions to treat exclusively non-infected patients [45]. This may not be feasible in some regions or districts and in such a case, the same hospital should be divided into areas or wards according to the risk of exposure to the virus, with a stratified increase in PPE according to the increase in the level of probable exposure (Figure 2) [45].

On the personnel level, all should apply the general self-protection rules like, safe distancing, face masks, goggles for eye protection, hand disinfection, regular decontamination of all patient/staff contact points, and avoidance of touching one’s eyes, nose, and face [49, 50]. Another notable recommendation is to establish a three-team approach where one team is working in the hospital involved in direct patient care, while the other teams are away from the hospital through 14 days as a “quarantine” between episodes of direct patient care [51]. This requires adequate number of medical staff which has not always been the case in the COVID-19 pandemic.

On the patient level, in hospitals still running outpatient clinics, patients should be screened for symptoms like (fever, cough, sore throat), if the patient shows positive symptoms consider delaying the outpatient management till test results are available. If a patient’s operation could not be delayed for testing, then the patient should be re-triaged into the emergency category and presumed COVID positive. All of this should be done with proper precautions such as wearing face masks, distancing, and well-aerated waiting areas [52, 53].

As for patients in the Emergency Department (ED) and crowded triage areas, same protective precautions apply, and if the patient is oriented, he/she is asked for the suspect criteria for COVID-19 infection. Suspected or confirmed COVID-19 patients should be isolated in a separate room and should keep at least 6 feet distance from other patients or non-treating staff. COVID-19 Polymerase Chain Reaction (PCR) testing should be done for all patients that will be admitted or will undergo surgery. Surgeons should not approach the triage area without the minimum standard PPE recommended. PPE should be exchanged if they are damaged or soiled or before leaving the ED. Only required equipment and assessment tools should be brought into the triage room to minimize the number of items that need to be disinfected after the exposure [45–54].

#### F. What are the peri-operative precautions when operating on a COVID-19 positive or suspected case?

This should be incorporated into hospital plans and rules to face the pandemic, [55] and can be subdivided into measures involving the operating room, personnel, anesthesia, the procedure, and postoperative precautions.

##### Operating room measures

Separate operating rooms (OR) should be designated for COVID-19 positive patients, isolated from other operating rooms [5–45]. The operating room is preferred to have a separate ventilation system with negative pressure [5, 58–60], which if not available, it is recommended to add High-Efficiency Particulate Air (HEPA) filters to positive pressure rooms [51–54]. Moreover, Air conditioning should be turned off [59–62].

Only the materials necessary for the case should be brought into the OR [5]. All equipment and screens should be covered with plastic sheets to facilitate decontamination [45]. Consider attenuation of residual environmental contamination through cleaning with surface disinfectants and ultraviolet light (UV-C) [63].

All traffic in and out of the OR should be minimized [5–54]. All doors should be closed once the patient is transferred in and during the whole operation [5]. The path of the patient to and from the OR should be kept clear and better to be separate from other operating rooms [5]. Patients should cover their face with a surgical mask [5]. The patient should recover in the operating room and transferred directly to the isolation ward [5–62].

The number of personnel inside the OR should be kept to the minimum. Services personnel should not enter the room until enough time has elapsed for air changers to reduce the risk of contamination [45–61]. Sales representatives, residents, and fellows should be discarded from OR unless essential [5–51].

##### Operating personnel

The fewest number of personnel possible is the main goal, with the highest skilled surgeon performing the procedure to avoid prolongation of the surgery [51, 54, 59, 64]. All personnel in the operating room should wear the PPE which include Association of Advancement of Medical Instrumentation (AAMI) level III surgical gowns, surgical hood (for head and neck covering), double gloves, facemasks and either N95, Filtering Face Piece 2 (FFP2) respirators with a face shield/googles or Powered Air-Purifying Respirator (PARP), fluid-resistant shoes or booties [5, 49–57]. Donning and doffing of PPE should be done in an anteroom if available, [5–56] with hand hygiene prior and after donning/doffing PPE [5]. Avoid self-contamination during PPE doffing. Disinfect the first pair of gloves with an alcohol solution, before removing the surgical mask with the shield and the hair cap [5–56]. Consider placing a simple surgical mask on top of the N-95 to prevent gross contamination. Each time N95 respirator is taken off, it must be double-checked for not being soiled or damaged before reuse [5, 45–57]. Full face shield is preferred to protective eye goggles [5, 52–57].

##### Anesthesia

Dedicated anesthesia machines should be exclusively designated for COVID-19 positive cases [62]. The most experienced anesthesiologist should intubate the patient in the shortest possible time with minimal airway manipulation, avoiding face mask ventilation and open-air way suction as possible [5, 45–60]. Keep the minimum number of personnel inside the anesthesia room which should be separate from the operating room, which should not be entered for 15–20 min after intubation [5, 51–57].

Use deep anesthesia and neuromuscular blockage. Pre-oxygenation should be performed via well-fitting face mask to avoid hypoxia in critically ill COVID-19 patients with respiratory failure [60–65]. It is preferred to avoid general anesthesia and use of regional/spinal anesthesia is recommended whenever possible [60].

##### Surgical procedure

Consider the use of minimally invasive approaches to decrease operating staff exposure and shorten case duration [5, 51–66]. Use disposable medical supplies/instruments whenever possible, and absorbable sutures for wound closure to avoid a postoperative unnecessary visit [45–51]. The use of electrocautery should be reduced to minimize the surgical smoke and should be used in conjunction with a smoke evacuator [5, 58, 59, 67]. Care should be taken when using sharp objects to avoid sharp injury or damage of PPE [61]. The use of power tools like bone saws, reamers, and drills should be reduced to the minimum and the power settings should be as low as possible, as they release aerosols, increasing the risk of virus spread. Suction devices to remove smoke and aerosols should be used during their use [68].

All body fluids as blood, secretions, urine, or pathological specimens should be collected in double sealed bags for inspection or destruction [5, 45–61]. All contaminated instruments and devices should be disinfected separately followed by proper labeling [5, 45–61].

##### Postoperative precautions

The transfer to isolation wards should be through dedicated corridors and elevators which should be carefully sterilized after transport [5 5, –62]. During the transfer, transport personnel should wear PPE which should not be the same as worn during the procedure and patients should be wearing N-95/FFP2 masks and covered with disposable operating sheets [5, 58–62].

Surgeons must be aware of common postoperative complications from COVID-19 infections. In the presence of fever and one of the symptoms of a respiratory infection (dry cough, etc.), laboratory tests for COVID-19 diagnosis must be ordered. Suspected cases should be reported immediately together with transfer of the patient to an isolation ward [69]. Patients should receive adequate nutrition, fluid hydration, and electrolyte balance to promote immune recovery and rapid rehabilitation [61]. Frequent monitoring of temperature, laboratory Complete Blood Count (CBC), C-reactive protein, and Ferritin level should be done [70]. Severe COVID-19 infection might cause a “cytokine storm syndrome”, which is characterized by a fulminant and fatal hyper-cytokinemia with multiorgan failure. An increased level of ferritin occurs in approximately 50% of patients. All patients with severe COVID-19 should be screened for hyper-inflammation markers [71].

##### Safe and effective patient care during the pandemic and telemedicine

In order to resume safe patient care, telemedicine has been widely used during this pandemic [72–75]. Telemedicine allows health care providers to deliver clinical services to patients through the use of the widely available telecommunication technologies. It can be used for patient triage, postoperative follow-up and monitoring patients with chronic diseases. Postoperative rehabilitation can be also resumed remotely via online educational programs or videoconferences. Moreover, rehabilitation can be tele-monitored through special technologies such as wireless sensors for range of motion such as the knee following knee arthroplasty. Nonetheless, telemedicine has its limitations. Patients with sutures to remove, cast to change or need comprehensive clinical examination will still have to pay an in person visit to the health care facility. Also, there are obstacles for wide implementation of such services such as infrastructure cost, provider and patient education, data protection, ethical consideration, legalization, and payment regulation [64].

In order to facilitate the use of telemedicine, the Office for Civil Rights at the U.S. Department of Health and Human Services on March 17, 2020 allowed physicians to utilize commercially available platforms, such as, Skype, WhatsApp, Zoom, and FaceTime without imposing penalties for noncompliance. In all cases, documentation within the patient medical record is mandatory [76].

##### Training during the pandemic

The COVID-19 crisis called for alternative methods to resume resident and fellow education [77–79], an example is the flipped virtual classroom method, in which the learners are asked to review the lecture online, with a subsequent virtual meeting focused on active learning and case-based discussions. Other methods include online practice questions, academic webinars, and telehealth clinics with resident involvement. Many applications such as Webex, Google Classroom, Microsoft Teams, and Zoom offer platforms for remote online conferences. The main drawback to this approach is that it cannot involve actual clinical or surgical skills teaching. Others include difficulties some senior staff may have with utilizing modern technology, slow internet speed in some regions, and difficulties with viewing some pictures especially radiology [80]. To overcome these obstacles high-definition 3D operative videos and surgical simulations are being employed. Various simulation modalities are available including surgical skills laboratories, cadaveric dissections and procedural training, and computer-based virtual reality training [81–84].

The 2020 Annual Meeting of American Academy of Orthopedic Surgeons (AAOS) is now being made available through the AAOS website. This includes instructional course lectures, and ask expert sessions, in addition to the traditional research paper and poster presentations. The American Association of Hip and Knee Surgeons (AAHKS) has developed the FOCAL Initiative: Fellows Online COVID-19 AAHKS Learning, a series of online lectures by invited faculty to continue fellow education during this time [81]. Follow-up of the online training has to be followed up by the person responsible for resident and fellow training through recording attendance and completion of online sessions and modules, and completing online assessments and quizzes [81].

##### Making use of the crisis and planning for the post-pandemic era

Global cooperation and exchange of experiences are still to be improved. The pandemic is still in various stages in different countries. Countries with increasing numbers of cases and mortalities are learning from those recovering from the crisis. The future requires better planning and re-allocation of resources to be prepared for such events.

To make use of the available technologies in telemedicine is of utmost importance. Remote triaging and examination techniques, feedback through mobile applications, and virtual interdisciplinary meetings should be encouraged. Urgent legislative reforms to adapt to these changes are mandatory.

E-learning, virtual conferences, webinars, and simulation training initiatives must be supported by the international scientific societies. Curricula should be revised to adapt to these needs. Non-technical skills should constitute integral part of learning programs.

Plans should be prepared to manage the accumulated long waiting lists of elective surgeries [85–89]. Prioritization should be rational without inferring excessive burden on the recovering health system after the crisis.

All in all, the authors believe that the medical practice, including orthopedic surgery will differ after the pandemic is over. Implementation of new technologies, restructuring our health systems with incorporation of telemedicine as well as reorganizing of our traditional training programs will be crucial for a more effective and optimal delivery of care.

## Conflict of Interest

The authors declare that they have no conflict of interest related to the submitted article.

There is no funding source.

This article does not contain any studies with human participants or animals performed by any of the authors.

## References

[1] Holshue ML, DeBolt C, Lindquist S, et al. (2020) First case of 2019 novel coronavirus in the United States. N Engl J Med 382, 929–936. 32004427 10.1056/NEJMoa2001191 PMC7092802

[2] Li Q, Guan X, Wu P, et al. (2020) Early transmission dynamics in Wuhan, China, of novel coronavirus-infected pneumonia. N Engl J Med 382, 1199–1207. 31995857 10.1056/NEJMoa2001316 PMC7121484

[3] Sun P, Lu X, Xu C, et al. (2020) Understanding of COVID-19 based on current evidence. J Med Virol 92, 548–551. 32096567 10.1002/jmv.25722 PMC7228250

[4] Houdek MT, Wagner ER, Wyles CC, et al. (2015) New options for vascularized bone reconstruction in the upper extremity. Semin Plast Surg 29, 20–29. 25685100 10.1055/s-0035-1544167 PMC4317278

[5] Brindle M, Gawande A (2020) Managing COVID-19 in surgical systems. Ann Surg 272, e1–e2. 32209891 10.1097/SLA.0000000000003923 PMC7188040

[6] Moher D, Liberati A, Tetzlaff J, et al. (2009) Preferred reporting items for systematic reviews and meta-analyses: The PRISMA statement. PLoS Med 6, e1000097. 19621072 10.1371/journal.pmed.1000097 PMC2707599

[7] Correia M, Ramos RF, Bahten LCV (2020) The surgeons and the COVID-19 pandemic. Rev Col Bras Cir 47, e20202536. 32236295 10.1590/0100-6991e-20202536

[8] Vaccaro AR, Getz CL, Cohen BE, et al. (2020) Practice management during the COVID-19 pandemic. J Am Acad Orthop Surg 28, 464–470. 32287086 10.5435/JAAOS-D-20-00379 PMC7197337

[9] Buerhaus PI, Auerbach DI, Staiger DO (2020) Older clinicians and the surge in novel coronavirus disease 2019 (COVID-19). JAMA. 10.1001/jama.2020.4978 32227200

[10] Dunham AM, Rieder TN, Humbyrd CJ (2020) A bioethical perspective for navigating moral dilemmas amidst the COVID-19 pandemic. J Am Acad Orthop Surg 28, 471–476. 32282442 10.5435/JAAOS-D-20-00371 PMC7197334

[11] Johns Hopkins Bloomberg School of Public Health CfHS (2010) Ventilator stockpiling and availability in the US. http://www.centerforhealthsecurity.org/resources/COVID-19/200214-VentilatorAvailability-factsheet.pdf. Accessed 10 April 2010.

[12] McBride KE, Brown KGM, Fisher OM, et al. (2020) Impact of the COVID-19 pandemic on surgical services: Early experiences at a nominated COVID-19 centre. ANZ J Surg 90, 663–665. 32259337 10.1111/ans.15900 PMC7262155

[13] Ranney ML, Griffeth V, Jha AK (2020) Critical supply shortages – the need for ventilators and personal protective equipment during the COVID-19 pandemic. N Engl J Med 382, e41. 32212516 10.1056/NEJMp2006141

[14] Truog RD, Mitchell C, Daley GQ (2020) The toughest triage – allocating ventilators in a pandemic. N Engl J Med 382, 1973–1975 10.1056/NEJMp2005689 32202721

[15] Prevention CfDCa (2020) Strategies for optimizing the supply of N95 respirators. https://www.cdc.gov/coronavirus/2019-ncov/hcp/respirators-strategy/index.html Accessed 04/20/2020 2020

[16] Vaishya R, Vaish A (2020) Roles and responsibilities of the orthopaedic community and the society during COVID-19 pandemic. Indian J Orthop 54, 398–399. 32296249 10.1007/s43465-020-00105-7 PMC7155759

[17] Boškoski I, Gallo C, Wallace MB, et al. (2020) COVID-19 pandemic and personal protective equipment shortage: Protective efficacy comparing masks and scientific methods for respirator reuse. Gastrointest Endosc S0016-5107(20)34247-4. 10.1016/j.gie.2020.04.048 PMC7184993 32353457

[18] Lancaster EM, Sosa JA, Sammann A, et al. (2020) Rapid response of an academic surgical department to the COVID-19 pandemic: Implications for patients, surgeons, and the community. J Am Coll Surg 230, 1064–1073. 10.1016/j.jamcollsurg.2020.04.007 PMC7194622 32278726

[19] Enriquez P (2016) Crispr-mediated epigenome editing. Yale J Biol Med 89, 471–486. 28018139 PMC5168826

[20] Zhang F Zhang (2017) Lab General Cloning Protocol. https://media.addgene.org/cms/filer_public/e6/5a/e65a9ef8-c8ac-4f88-98da-3b7d7960394c/zhang-lab-general-cloning-protocol.pdf.

[21] Ashford RU, Nichols JS, Mangwani J (2020) Annotation: The COVID-19 pandemic and clinical orthopaedic and trauma surgery. J Clin Orthop Trauma 11, 504–505. 32292257 10.1016/j.jcot.2020.04.002 PMC7132434

[22] Ducournau F, Arianni M, Awwad S, et al. (2020) Covid- 19: Initial experience of an International Group of Hand Surgeons. Hand Surg Rehabil 39, 159–166. 32278932 10.1016/j.hansur.2020.04.001 PMC7194873

[23] Ghogawala Z, Kurpad S, Falavigna A, et al. (2020) Editorial. COVID-19 and spinal surgery. J Neurosurg Spine, 1–3. 10.3171/2020.4.SPINE20468 PMC7164398 32302989

[24] Peloso A, Moeckli B, Oldani G, et al. (2020) Response of a European surgical department to the COVID-19 crisis. Swiss Med Wkly 150, w20241. 32271935 10.4414/smw.2020.20241

[25] Thaler M, Khosravi I, Hirschmann MT, et al. (2020) Disruption of joint arthroplasty services in Europe during the COVID-19 pandemic: An online survey within the European Hip Society (EHS) and the European Knee Associates (EKA). Knee Surg Sports Traumatol Arthrosc, 1–8. 10.1007/s00167-020-06033-1 PMC7195619 32361927

[26] D’Apolito R, Faraldi M, Ottaiano I, et al. (2020) Disruption of arthroplasty practice in an orthopedic center in northern Italy during the coronavirus disease 2019 pandemic. J Arthroplasty 35, S6–S9. 10.1016/j.arth.2020.04.057 PMC7194696 32370923

[27] Meneghini RM (2020) Resource reallocation during the COVID-19 pandemic in a suburban hospital system: Implications for outpatient hip and knee arthroplasty. J Arthroplasty 35, S15–S18. 10.1016/j.arth.2020.04.051 PMC7175890 32376170

[28] Ahmed S, Leong Glenn TW, Chong YL (2020) Surgical response to COVID-19 pandemic: A Singapore perspective. J Am Coll Surg 230, 1074–1077. 10.1016/j.jamcollsurg.2020.04.003 PMC7194919 32278729

[29] Haleem A, Javaid M, Vaishya R, et al. (2020) Effects of COVID-19 pandemic in the field of orthopaedics. J Clinic Orthop Trauma 11, 498-499. 10.1016/j.jcot.2020.03.015 PMC7211812 32405218

[30] Ross SW, Lauer CW, Miles WS, et al. (2020) Maximizing the calm before the storm: Tiered surgical response plan for novel coronavirus (COVID-19). J Am Coll Surg 230, 1080–1091.e3. 10.1016/j.jamcollsurg.2020.03.019 PMC7128345 32240770

[31] Sarpong NO, Forrester LA, Levine WN (2020) What’s important: Redeployment of the orthopaedic surgeon during the COVID-19 pandemic: Perspectives from the trenches. J Bone Joint Surg Am 102, 1019–102. 10.2106/JBJS.20.00574 PMC7224625 32287087

[32] Xu J, Xu QH, Wang CM, et al. (2020) Psychological status of surgical staff during the COVID-19 outbreak. Psychiatry Res 288, 112955. 32302815 10.1016/j.psychres.2020.112955 PMC7151272

[33] Khazaei M, Asgari R, Zarei E, et al. (2020) Incidentally diagnosed COVID-19 infection in trauma patients; a clinical experience. Arch Acad Emerg Med 8, e31. 32232216 PMC7092921

[34] Mi B, Chen L, Xiong Y, et al. (2020) Characteristics and early prognosis of COVID-19 infection in fracture patients. J Bone Joint Surg Am 102, 750–758. 10.2106/JBJS.20.00390 PMC7219849 32379114

[35] Catellani F., Coscione A., D’Ambrosi R., et al. (2020) Treatment of proximal femoral fragility fractures in patients with COVID-19 during the SARS-CoV-2 outbreak in northern Italy. JBJS; Latest Articles. 10.2106/JBJS.20.00617 PMC7224593 32345864

[36] Chen YC, Lin WC (2017) Risk of long-term infection-related death in clinical osteoporotic vertebral fractures: A hospital-based analysis. PLoS One 12, e0182614. 28793342 10.1371/journal.pone.0182614 PMC5549923

[37] Stahel PF (2020) How to risk-stratify elective surgery during the COVID-19 pandemic? Patient Saf Surg 14, 8. 32288785 10.1186/s13037-020-00235-9 PMC7107008

[38] Sarac NJ, Sarac BA, Schoenbrunner AR, et al. (2020) A review of state guidelines for elective orthopaedic procedures during the COVID-19 outbreak. J Bone Joint Surg Am 102, 942–945. 10.2106/JBJS.20.00510 PMC7197340 32282419

[39] Lee J, Choi JY, Kim MS (2020) Elective surgeries during the COVID-19 outbreak. British Journal of Surgery. 10.1002/bjs.11697 PMC7273018 32406928

[40] Besnier E, Tuech JJ, Schwarz L (2020) We asked the experts: COVID-19 outbreak: Is there still a place for scheduled surgery? “Reflection from pathophysiological data”. World J Surg. 10.1007/s00268-020-05501-6 PMC7124188 32246185

[41] Lei S, Jiang F, Su W, et al. (2020) Clinical characteristics and outcomes of patients undergoing surgeries during the incubation period of COVID-19 infection. EClinicalMedicine, 100331. 32292899 10.1016/j.eclinm.2020.100331 PMC7128617

[42] Farrell S, Schaeffer EK, Mulpuri K (2020) Recommendations for the care of pediatric orthopedic patients during the COVID pandemic. J Am Acad Orthop Surg 28, e477–e486. 10.5435/JAAOS-D-20-00391 PMC7197339 32301817

[43] DePhillipo NN, Larson CM, O’Neill OR, et al. (2020) Guidelines for ambulatory surgery centers for the care of surgically necessary/time-sensitive orthopaedic cases during the COVID-19 pandemic. J Bone Joint Surg Am. 10.2106/JBJS.20.00489 PMC7219855 32282420

[44] Massey PA, McClary K, Zhang AS, et al. (2020) Orthopaedic surgical selection and inpatient paradigms during the coronavirus COVID-19 pandemic. J Am Acad Orthop Surg. 10.5435/JAAOS-D-20-00360 PMC7195848 32304401

[45] Awad ME, Rumley JCL, Vazquez JA, et al. (2020) Peri-operative considerations in urgent surgical care of suspected and confirmed COVID-19 orthopedic patients: Operating rooms protocols and recommendations in the current COVID-19 pandemic. J Am Acad Orthop Surg. 10.5435/JAAOS-D-20-00227 PMC7197335 32282441

[46] Balibrea JM, Badia JM, Rubio Perez I, et al. (2020). Surgical management of patients with COVID-19 infection. Recommendations of the Spanish Association of Surgeons Cir Esp.10.1016/j.ciresp.2020.03.001PMC727042832252979

[47] De Vitis R, Passiatore M, Perna A, et al. (2020) COVID-19 contagion and contamination through hands of trauma patients: What risks and what precautions? J Hosp Infect. 10.1016/j.jhin.2020.03.037 PMC7129819 32259547

[48] Donnally CJ 3rd, Shenoy K, Vaccaro AR, et al. (2020) Triaging spine surgery in the COVID-19 era. Clin Spine Surg. 10.1097/BSD.0000000000000988 PMC7172569 32235170

[49] Viswanath A, Monga P (2020) Working through the COVID-19 outbreak: Rapid review and recommendations for MSK and allied heath personnel. J Clin Orthop Trauma. 10.1016/j.jcot.2020.03.014 PMC7102609 32292256

[50] Yeo D, Yeo C, Kaushal S, et al. (2020) COVID-19 & the general surgical department – measures to reduce spread of SARS-Cov-2 among surgeons. Ann Surg. 10.1097/SLA.0000000000003957 PMC7188025 32301805

[51] Stinner DJ, Lebrun C, Hsu JR, et al. (2020) The orthopaedic trauma service and COVID-19 – practice considerations to optimize outcomes and limit exposure. J Orthop Trauma. 10.1097/BOT.0000000000001782 PMC7188036 32301767

[52] Forrester JD, Nassar AK, Maggio PM, et al. (2020) Precautions for operating room team members during the COVID-19 pandemic. J Am Coll Surg. 10.1016/j.jamcollsurg.2020.03.030 PMC7270564 32247836

[53] Kmietowicz Z (2020) Rules on isolation rooms for suspected COVID-19 cases in GP surgeries to be relaxed. BMJ (Clinical research ed.) 368, m707. 10.1136/bmj.m707 32086235

[54] Vannabouathong C, Devji T, Ekhtiari S, et al. (2020) Novel coronavirus COVID-19: Current evidence and evolving strategies. J Bone Joint Surg Am. 10.2106/JBJS.20.00396 PMC7219842 32379112

[55] Aminian A, Safari S, Razeghian-Jahromi A, et al. (2020) COVID-19 outbreak and surgical practice: Unexpected fatality in perioperative period. Ann Surg. 10.1097/SLA.0000000000003925 PMC7188030 32221117

[56] Rodrigues-Pinto R, Sousa R, Oliveira A (2020) Preparing to perform trauma and orthopaedic surgery on patients with COVID-19. J Bone Joint Surg Am. 10.2106/JBJS.20.00454 PMC7197341 32282412

[57] Abdelrahman T, Ansell J, Brown C, et al. (2020) Systematic review of recommended operating room practice during the COVID-19 pandemic. BJS Open. 10.1002/bjs5.50304 PMC7272923 32395909

[58] Chow TT, Yang XY (2004) Ventilation performance in operating theatres against airborne infection: Review of research activities and practical guidance. J Hosp Infect 56, 85–92. 15019218 10.1016/j.jhin.2003.09.020

[59] Ti LK, Ang LS, Foong TW, et al. (2020) What we do when a COVID-19 patient needs an operation: Operating room preparation and guidance. Can J Anaesth. 10.1007/s12630-020-01617-4 PMC7090746 32144591

[60] Wax RS, Christian MD (2020) Practical recommendations for critical care and anesthesiology teams caring for novel coronavirus (2019-nCoV) patients. Can J Anaesth 67, 568–576. 32052373 10.1007/s12630-020-01591-x PMC7091420

[61] Chandy PE, Nasir MU, Srinivasan S, et al. (2020) Interventional radiology and COVID-19: Evidence-based measures to limit transmission. Diagn Interv Radiol. 10.5152/dir.2020.20166 PMC7239364 32229433

[62] Chen X, Liu Y, Gong Y, et al. (2020) Perioperative management of patients infected with the novel coronavirus: Recommendation from the joint task force of the Chinese Society of Anesthesiology and the Chinese Association of Anesthesiologists. Anesthesiology. 10.1097/ALN.0000000000003301 PMC7155907 32195699

[63] Dexter F, Parra MC, Brown JR, et al. (2020) Perioperative COVID-19 defense: An evidence-based approach for optimization of infection control and operating room management. Anesth Analg. 10.1213/ANE.0000000000004829 PMC7172574 32217947

[64] Chang Liang Z, Wang W, Murphy D, et al. (2020) Novel coronavirus and orthopaedic surgery: Early experiences from Singapore. J Bone Joint Surg Am. 10.2106/JBJS.20.00236 PMC7141583 32379113

[65] Chee VW, Khoo ML, Lee SF, et al. (2004) Infection control measures for operative procedures in severe acute respiratory syndrome-related patients. Anesthesiology 100, 1394–1398. 15166557 10.1097/00000542-200406000-00010

[66] Chadi SA, Guidolin K, Caycedo-Marulanda A, et al. (2020) Current evidence for minimally invasive surgery during the COVID-19 pandemic and risk mitigation strategies: A narrative review. Ann Surg. 10.1097/SLA.0000000000004010 PMC7268822 32675513

[67] Zheng MH, Boni L, Fingerhut A (2020) Minimally invasive surgery and the novel coronavirus outbreak: Lessons learned in China and Italy. Ann Surg. 10.1097/SLA.0000000000003924 PMC7188059 32221118

[68] Yeh HC, Turner RS, Jones RK, et al. (1995) Characterization of aerosols produced during surgical procedures in hospitals. Aero Sci Technol 22, 151–161.

[69] BJS Society (2020) Global guidance for surgical care during the COVID-19 pandemic. Br J Surg. 10.1002/bjs.11646 PMC7262310 32293715

[70] Seguin A, Galicier L, Boutboul D, et al. (2016) Pulmonary involvement in patients with hemophagocytic lymphohistiocytosis. Chest 149, 1294–1301. 26836913 10.1016/j.chest.2015.11.004

[71] Mehta P, McAuley DF, Brown M, et al. (2020) COVID-19: Consider cytokine storm syndromes and immunosuppression. Lancet 395, 1033–1034. 32192578 10.1016/S0140-6736(20)30628-0 PMC7270045

[72] Parisien R.L., Shin M., Constant M., et al. (2020) Telehealth utilization in response to the novel coronavirus (COVID-19) pandemic in orthopaedic surgery. J Am Acad Orthop Surg. Publish Ahead of Print. 10.5435/JAAOS-D-20-00339 PMC7197336 32459409

[73] Tanaka MJ, Oh LS, Martin SD, et al. (2020) Telemedicine in the era of COVID-19: The virtual orthopaedic examination. J Bone Joint Surg Am. 10.2106/JBJS.20.00609 PMC7224627 32341311

[74] Pratap Singh R, Javaid M, Haleem A, et al. (2020) Internet of medical things (iomt) for orthopaedic in COVID-19 pandemic: Roles, challenges, and applications. J Clin Orthop Trauma. 10.1016/j.jcot.2020.05.011 PMC7227564 32425428

[75] Bini SA, Schilling PL, Patel SP, et al. (2020) Digital orthopaedics: A glimpse into the future in the midst of a pandemic. J Arthroplasty. 10.1016/j.arth.2020.04.048 PMC7175889 32416956

[76] Halawi MJ, Wang DD, Hunt TR 3rd (2020) What’s important: Weathering the COVID-19 crisis: Time for leadership, vigilance, and unity. J Bone Joint Surg Am. 10.2106/JBJS.20.00419 PMC7219834 32379115

[77] Dowdell JE, Louie PK, Virk S, et al. (2020) Spine fellowship training reorganizing during a pandemic: Perspectives from a tertiary orthopedic specialty center in the epicenter of outbreak. Spine J. 10.1016/j.spinee.2020.04.015 PMC7194823 32344060

[78] Tait S, MacLean R, Gopinath B (2020) Covid 19 and training in the UK – correspondence. Int J Surg. 10.1016/j.ijsu.2020.04.061 PMC7252022 32371151

[79] Saleh H. (2020) Orthopedic surgery training during the COVID-19 pandemic: Dusting off my stethoscope. Spine (Phila Pa 1976). 10.1097/BRS.0000000000003561 PMC7299105 32404860

[80] Chick RC, Clifton GT, Peace KM, et al. (2020) Using technology to maintain the education of residents during the COVID-19 pandemic. J Surg Educ. 10.1016/j.jsurg.2020.03.018 PMC7270491 32253133

[81] Kogan M, Klein SE, Hannon CP, et al. (2020) Orthopaedic education during the COVID-19 pandemic. J Am Acad Orthop Surg. 10.5435/JAAOS-D-20-00292 PMC7195844 32282439

[82] McKechnie T, Levin M, Zhou K, et al. (2020) Virtual surgical training during COVID-19: Operating room simulation platforms accessible from home. Ann Surg. 10.1097/SLA.0000000000003999 PMC7268842 32675522

[83] Day RW, Taylor BM, Bednarski B, et al. (2020) Virtual interviews for surgical training program applicants during COVID-19: Lessons learned and recommendations. Ann Surg. 10.1097/SLA.0000000000004064 PMC7268876 32675519

[84] Ehrlich H, McKenney M, Elkbuli A (2020) We asked the experts: Virtual learning in surgical education during the COVID-19 pandemic-shaping the future of surgical education and training. World J Surg 1–3. 32409866 10.1007/s00268-020-05574-3 PMC7224589

[85] Oussedik S, Zagra L, Shin GY, et al. (2020) Reinstating elective orthopaedic surgery in the age of COVID-19. Bone Joint J 1–4. 10.1302/0301-620X.102B7.BJJ-2020-0808 32412313

[86] Parvizi J, Gehrke T, Krueger CA, et al. (2020) Resuming elective orthopaedic surgery during the COVID-19 pandemic: Guidelines developed by The International Consensus Group (ICM). J Bone Joint Surg Am. 10.2106/JBJS.20.00844 PMC7431146 32675662

[87] O’Connor CM, Anoushiravani AA, DiCaprio MR, et al. (2020) Economic recovery after the COVID-19 pandemic: Resuming elective orthopedic surgery and total joint arthroplasty. J Arthroplasty. 10.1016/j.arth.2020.04.038 PMC7166028 32345566

[88] Mouton C, Hirschmann MT, Ollivier M, et al. (2020) COVID-19 – ESSKA guidelines and recommendations for resuming elective surgery. J Exp Orthop 7, 28. 32405872 10.1186/s40634-020-00248-4 PMC7220621

[89] de Caro F, Hirschmann TM, Verdonk P (2020) Returning to orthopaedic business as usual after COVID-19: Strategies and options. Knee Surg Sports Traumatol Arthrosc 1–6.

